# Genetic Diversity and DNA Barcoding of Thrips in Bangladesh

**DOI:** 10.3390/insects15020107

**Published:** 2024-02-03

**Authors:** Mst. Fatema Khatun, Hwal-Su Hwang, Jeong-Hun Kang, Kyeong-Yeoll Lee, Eui-Joon Kil

**Affiliations:** 1 Department of Plant Medicals, Andong National University, Andong 36729, Republic of Korea; k.fatemabsmrau@gmail.com (M.F.K.); wjdgns3771@naver.com (J.-H.K.); 2 Agricultural Science and Technology Research Institute, Andong National University, Andong 36729, Republic of Korea; 3 Department of Entomology, Bangabandhu Sheikh Mujibur Rahman Agricultural University, Gazipur 1706, Bangladesh; 4 Department of Plant Medicine, College of Agriculture and Life Science, Kyungpook National University, Daegu 37224, Republic of Korea; bgtwo2@naver.com (H.-S.H.); leeky@knu.ac.kr (K.-Y.L.); 5 Institute of Plant Medicine, Kyungpook National University, Daegu 37224, Republic of Korea; 6 Institute of Agricultural Science and Technology, Kyungpook National University, Daegu 37224, Republic of Korea

**Keywords:** diversity, geographic populations, genetic structure, haplotypes, interspecific, mitochondrial COI

## Abstract

**Simple Summary:**

Thrips, the notorious sap-sucking insects, serve as the vector of plant viruses. Their accurate species identification is essential for determining the vector species and implementing successful pest management techniques. Mitochondrial COI (DNA barcode) sequence variation has proven to be effective in identifying species in many insect pest groups. In this study, we identified 19 thrips species that were found on different host plants in Bangladesh. Among the 19 species, we identified four prominent vector species (*Frankliniella intonsa*, *Thrips tabaci*, *Scirtothrips dorsalis* and *T. palmi*) and one significant pollinator, *Microcephalothrips abdominalis*. The findings presented here emphasize the importance of conducting DNA barcoding, analyzing population structure, and assessing the genetic diversity of thrips species in this region. This research is crucial for comprehending their host preferences, potential for adaptation, and genetic variation at both local and regional levels. Accurate identification of pests and invasive species is essential for implementing effective control and quarantine measures. Misidentifications can lead to the application of ineffective control strategies.

**Abstract:**

Thrips are economically important pests, and some species transmit plant viruses that are widely distributed and can damage vegetables and cash crops. Although few studies on thrips species have been conducted in Bangladesh, the variation and genetic diversity of thrips species remain unknown. In this study, we collected thrips samples from 16 geographical locations throughout the country and determined the nucleotide sequences of the mitochondrial cytochrome c oxidase subunit 1 (mtCOI) gene in 207 thrips individuals. Phylogenetic analysis revealed ten genera (*Thrips*, *Haplothrips*, *Megalothrips*, *Scirtothrips*, *Frankliniella*, *Dendrothripoides*, *Astrothrips*, *Microcephalothrips*, *Ayyaria,* and *Bathrips*) and 19 species of thrips to inhabit Bangladesh. Among these, ten species had not been previously reported in Bangladesh. Intraspecific genetic variation was diverse for each species. Notably, *Thrips palmi* was the most genetically diverse species, containing 14 haplotypes. The Mantel test revealed no correlation between genetic and geographical distances. This study revealed that thrips species are expanding their host ranges and geographical distributions, which provides valuable insights into monitoring the diversity of and control strategies for these pests.

## 1. Introduction

Thrips are serious sap-sucking insect pests and vectors in many economically important crops worldwide [1,2]. Many species of thrips are polyphagous, launching assaults both on perennial and annual crops, thereby enhancing their survival and propagation capabilities [3]. Thrips can drastically undermine crop productivity and nutritional security by either directly feeding on crops or indirectly transmitting pathogenic plant viruses. Orthotospoviruses are transmitted and spread in nature by vector insects belonging to the family Thripidae (Thysanoptera), genus *Frankliniella*, and *Thrips*. Fourteen species of thrips (Thripidae) are vectors of orthotospoviruses, and nearly 1% are pests of agricultural crops [4]. Thrips are the insect vectors of orthotospoviruses (genus *Orthotospovirus* and family *Tospoviridae*, and order *Bunyavirales*), which affect cultivated and wild plants in several unrelated plant families worldwide [5,6]. Among the known species of thrips (Thripidae); *Ceratothripoides claratris*, *Frankliniella bispinosa*, *F. cephalica*, *F. fusca*, *F. gemina*, *F. intonsa*, *F. occidentalis*, *F. schultzei*, *F. zucchini*, *Thrips flavus*, *T. palmi*, *T. setosus*, *T. tabaci*, *Scirtothrips dorsalis*, and *Dictyothrips betae* have been reported as vectors of plant pathogens [1,7,8,9,10,11,12]. Orthotospoviruses, including the tomato spotted wilt virus (TSWV, *Orthotospovirus tomatomaculae*), have become significant contributors to global economic losses in food and ornamental crops, with losses potentially reaching up to 100% [9,13,14]. At present, TSWV has an infection rate of 50-90% and is considered one of the top ten most economically detrimental plant viruses. It causes annual worldwide losses exceeding one billion dollars [15].

Identifying thrips at the species level is challenging because of several factors. These include their minuscule size, slight morphological differences, intraspecific polymorphisms, and sexual dimorphisms. This situation is further complicated by the presence of multiple species on the same host plant, significant intraspecific variations within thrips populations, and the requirement for specialized taxonomic knowledge [16,17,18,19]. Molecular identification systems are attractive because thrips are difficult to identify morphologically [20]. The molecular identification of thrips offers substantial benefits over morphology-based analysis. It effectively addresses the complexities arising from morphological variations across different life stages and the subtle morphological differences between species [16,17]. DNA barcodes have been providing accurate and rapid species identification since their inception. Barcode data on economically significant taxa, like Thysanoptera, can establish a common platform for diverse biological studies. These studies can encompass taxonomy, ecology, behavior, life history, pest management, and vector-virus relationships. Moreover, DNA barcode data can enhance taxonomic research by uncovering cryptic species, resolving species complexes, and assisting in the identification of new species [21,22]. The DNA barcoding technique provides molecular identification of thrips species [3,23,24,25], sex and polymorphisms [26], and the ability to discriminate between cryptic species [27,28], biotypes [29], haplotypes [30], and host and geographical genetic differences [6,17]. The mitochondrial cytochrome c oxidase subunit I gene (mtCOI) is the “DNA barcode”, showing 2% sequence divergence within species and >2% between species [31,32]. In addition, DNA barcoding can be used in insect pest management programs to determine the selection and timing of management practices based on polymorphisms and host adaptations [17,28].

According to previous studies, different genetic lineages and populations have varying abilities to transmit plant viruses [33,34,35,36,37,38], host plant preferences [35,39,40], and insecticide resistance [41]. In the natural environment, ecological and evolutionary dynamics are primarily influenced by two key factors: genetic diversity and population structure [42]. To ensure biodiversity conservation, all aspects of genetic diversity, including those of wild animals, insects, and agricultural crops, must be considered. Several parameters can be used to assess the degree to which an animal has a high level of genetic diversity efficiency and the subdivision of its population, such as the expected heterozygous number (He), observed heterozygosity (Ho), nucleic acid diversity (Pi), and polymorphism information content (PIC) [43].

In Bangladesh, it is common to grow vegetables, pulses, and flowers in both commercial fields and backyard gardens. However, crop production is severely hampered by insect infestation, which is one of the main causes of reduced yields [44]. Among the various insect pests that attack these crops, thrips are especially problematic. Thrips are widely distributed throughout Bangladesh and cause damage to many crops, such as onions, brinjals, ornamental flowers, chili, cotton, sweet potato, pumpkin, rose, marigold, gourds, beans, and tomatoes. Thrips can also transmit viral diseases to crops and cause significant yield losses. According to a survey conducted by the Asian Vegetable Research and Development Center in Asia, aphids, moths, and thrips are the most common vegetable pests. Particularly, during dry weather conditions, thrips exhibit heightened multiplication rates, contributing to yield losses ranging from approximately 50% to 90% [45]. In Bangladesh, production per unit area is relatively low due to insect pests, which generally cause 30–40% losses and sometimes 100% losses if no control measures are taken. Nevertheless, it varied from place to place and over time. In Bangladesh, farmers use synthetic insecticides extensively to combat thrips, spraying them more than once a week throughout the growing season. As a result of the indiscriminate use of pesticides, natural enemies are destroyed, beneficial arthropods are harmed, and eventually, they become resistant to those pesticides [46,47].

Few studies were done by other researchers [48] using Bangladeshi thrips samples from only bean host plants, but the genetic structure and variation of thrips populations in different host plants in Bangladesh remain unclear. In Bangladesh, the taxonomy and molecular identification of thrips species are not clearly defined; therefore, we conducted a study to identify thrips species in various host plants and gain some preliminary insight into their genetic and geographic diversity and their potential damage to crops. The objective of this study was to investigate the genetic variation and DNA barcoding of thrips species inhabiting various hosts across different regions in Bangladesh, utilizing mitochondrial DNA. The necessity to survey the DNA barcode, population structure, and genetic diversity of thrips species in this region stems from the need to comprehend host preferences, potential for adaptation, and genetic variation on both local and regional scales. This understanding is crucial for limiting the development and execution of new control strategies.

## 2. Materials and Methods

### 2.1. Thrips Collection

Thrips (adults and nymphs) were collected from various host plants (bean, garlic, rose, marigold, lemon, pumpkin, cucumber, mustard, brinjal, ash gourds, bitter gourd, sweet potato, ornamental flowers, chili, cotton, tomato, etc.) (Table 1) across 16 regions of Bangladesh from 2021 to 2023. Nymphs and adults were gathered on white paper utilizing a plant beating technique and subsequently preserved in separate vials containing 80% alcohol, categorized based on their respective locations and host plants. These vials were then stored at −20 °C pending further analysis (refer to Table S1). An estimated 20–150 thrips were collected from each site, with a minimum of three sites surveyed per crop field.

### 2.2. DNA Extraction

Genomic DNA from individual thrips was extracted using the PureLink^TM^ Genomic DNA Mini Kit (Invitrogen, Carlsbad, CA, USA). Each sample was placed into a 1.5 mL centrifuge tube containing 180 μL of digestion buffer and 20 μL of proteinase K (50 μg/mL), and then incubated at 55 °C for 4 hours. Following the manufacturer’s guidelines, DNA samples were extracted. The DNA concentration was measured using a NanoPhotometer^TM^ (Implen GmbH, Schatzbogen, Germany).

### 2.3. PCR Amplification

mtCOI DNA was amplified using the primer pair HCO-2198 and LCO-1490 [49]. The PCR process was conducted with a total reaction volume of 20 μL, which included 10 μL SmartGene 2× Dye Mixed Taq (SmartGENE, Daejeon, Republic of Korea), 1 μL of each primer (10 pmol/μL), 5 μL of nuclease-free water and 3 μL of template DNA solution (40 ng). The reaction mixtures underwent amplification with the following parameters: an initial denaturation at 94 °C for 5 min; this was followed by 35 cycles of 94 °C for 30 s, 52 °C for 30 s, and 72 °C for 60 s; and a final extension at 72 °C for 5 min. This process was carried out in a T100 Thermal Cycler from Bio-Rad (Hercules, CA, USA). The PCR products were electrophoresed on a 1% agarose gel, which was then purified using Expin^TM^ Gel SV (GeneAll, Seoul, Republic of Korea). The PCR amplicons were subcloned into a cloning vector (the pGEM-T Easy vector, Promega, Madison, WI, USA) and sequenced using Sanger sequencing (Macrogen, Seoul, Republic of Korea).

### 2.4. DNA Sequence and Barcoding Analysis

The GenBank database of the NCBI was searched using BLAST [50], and nucleotide sequence alignment was performed using CLUSTAL X1.83 [51]. The mtCOI sequences identified from Bangladeshi thrips samples were submitted to GenBank. The mean genetic distance among the samples was determined using MEGA 11.0 [52,53]. DNA barcoding [31] determines the genetic distance between samples based on the K2P distance [54] to reveal barcode gaps or breaks in the distribution of genetic distances between samples belonging to the same or different species [31,54]. The DNA barcode is derived from the sequence of the mtCOI gene. This sequence typically exhibits a 2% divergence between different species [31,32].

### 2.5. Phylogenetic Analysis

mtCOI sequences were manually edited at 655 bp for each thrips sample using CLC Genomics Workbench software (QIAGEN, Hilden, Germany). Multiple sequence alignments were performed using Clustal X 1.83 [51]. A phylogenetic tree was constructed using the Interactive Tree Of Life (iTOL v.6) software [55]. To test the robustness of the phylogeny, 1000 bootstrap replicates were used [56]. The *p*-distances were estimated using MEGA 11.0 (Table 2).

### 2.6. Population Structure and Genetic Analysis

The determination of genetic diversity parameters was carried out using DnaSP 5.12.01. This analysis included the determination of segregation sites, haplotypes, haplotype diversity, and nucleotide diversity. These parameters encompassed sequence polymorphisms, divergence, gene flow, neutrality tests (including Fu’s Fs and Tajima’s D), genetic differentiation, and pairwise Fst values [57,58]. The Mantel test was employed to ascertain the correlation between genetic distance (Fst) and geographic distance [59]. The geographic distances between the populations studied were based on the central location of the collection sites in Bangladesh. The sequences were examined using a minimum spanning network relationship among thrips species to create a haplotype network in PopART 1.7 [60]. The analysis of molecular variance (AMOVA) was substantiated with 1023 permutations [61]. AMOVA analysis and F-statistics of genetic variation for thrips populations in Bangladesh were derived using the pairwise distance method in Arlequin software version 3.5 [62]. The total molecular variance was categorized into ‘inter-group’ genetic variation (Fct), ‘intra-group’ genetic variation (Fsc), and ‘inter-population’ (Fst).

## 3. Results

### 3.1. Identification of Thrips Species in Bangladesh

**Figure 1:**
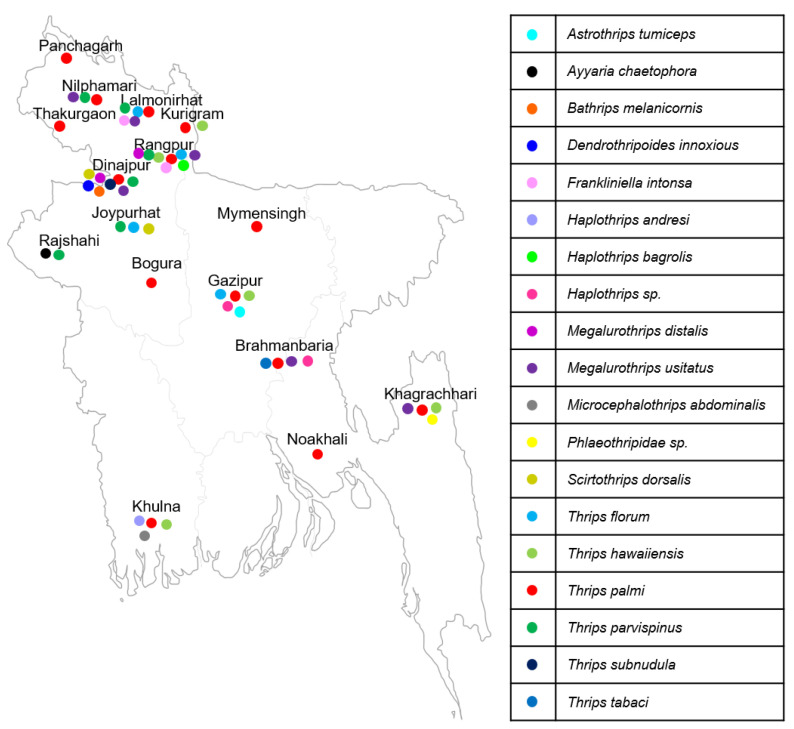
Sample collection sites and distribution of thrips species in Bangladesh. The colored symbols represent nineteen species.

**Figure 2:**
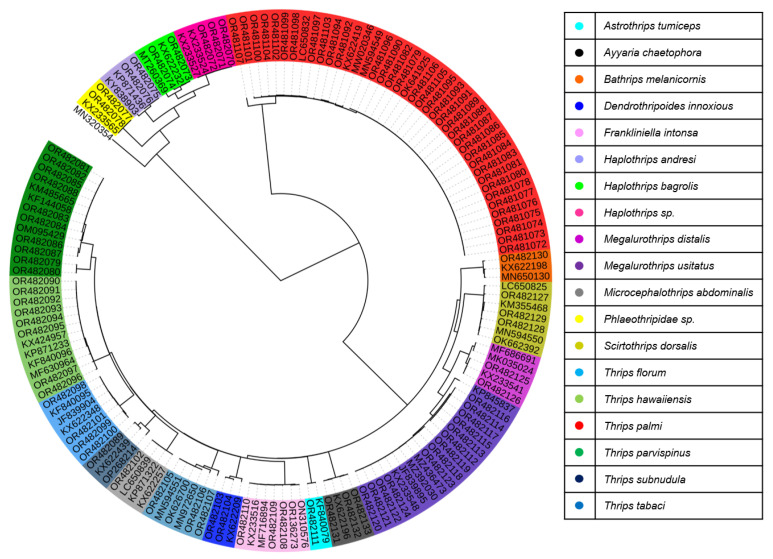
Phylogenetic tree based on mtCO1 sequences of thrips species in Bangladesh. The tree was generated based on the mtCOI sequences from the collected samples, and the related reference sequences were searched from the GenBank database in this study using Interactive Tree Of Life (iTOL) software. *Rhopalosiphum padi* (MN320354) was used as an outgroup.

A total of 207 mtCOI gene sequences were analyzed to study the genetic diversity and DNA barcoding of thrips species collected from 16 locations throughout Bangladesh between 2021 and 2023. The sequences have been submitted to the NCBI GenBank database and can be accessed using accession numbers OR481072–OR481107 and OR482070–OR482132 (Table S1 and Figure 1). Comparison of the sequences with those in NCBI GenBank and Barcode of Life Data System (BOLD) revealed close sequence matches range from (>97–100%) of these 19 species. Among all the samples, the mtCOI sequence variation ranged from 0.2 to 35.0%. (Table S3). Based on the phylogenetic tree of previously known sequences from the Genbank database, all samples were classified into 19 species across ten genera namely, *Thrips palmi*, *Bathrips melanicornis*, *Microcephalothrips abdominalis*, *T. tabaci*, *T. florum*, *Ayyaria chaetophora*, *T. hawaiiensis*, *T. subnudula*, *T. parvispinus*, *Haplothrips* sp., *H. bagrolis, H. andresi*, *Phlaeothripidae* sp., *Megalurothrips usitatus*, *M. distalis*, *Scirtothrips dorsalis*, *Dendrothripoides innoxius*, *Frankliniella intonsa*, and *Astrothrips tumiceps* (Figure 2, Table S1). A color-coded matrix showing pairwise identity scores between the nucleotide sequences of thrips species is presented in Figure S1. Among the 207 samples, *T. palmi* (47%) was the most abundant, while *M. usitatus* (14%), *T. parvispinus* (9%), *T. hawaiiensis* (9%), *Haplothrips* sp., *T. florum* and *F. intonsa* (3%), *A. chaetophora and S. dorsalis* (2%), *D. innoxius*, *H. bagrolis*, *H. andresi*, *Phlaeothripidae* sp., *T. tabaci*, and *M. distalis* (1%) had lower abundances. Notably, *M. abdominalis*, *B. melanicornis*, *A. tumiceps*, and *T. subnudula* were detected in only one sample (Table 1). We did not detect the *F. occidentalis* species in this study.

Geographic analysis showed that *T. palmi*, *T. hawaiiensis*, and *M. usitatus* were widely distributed throughout the country, whereas *T. parvispinus*, *S. dorsalis*, *F. intonsa*, *A. chaetophora*, *D. innoxious*, *B. melanicornis* and *T. subnudula* were found only in the northern part of Bangladesh. *T. florum* is distributed in the central and northern regions of Bangladesh, whereas *Haplothrips* sp., *A. tumiceps*, and *T. tabaci* are distributed in the central regions. *H. andresi*, *Phlaeothripidae* sp. *M. abdominalis* was found in Khulna and Khagrachori, located in southern Bangladesh (Figure 1).

Thrips samples were collected from 21 different crop species—bean (*Phaseolus vulgaris*), brinjal (*Solanum melongena*), cotton (*Gossypium hirsutum*), rose (*Rosa sciensis*), sponge gourd (*Luffa cylindrica*), bitter gourd (*Momordica charantia*), ash gourd (*Benincasa hispida*), ridge gourd (*Luffa acutangula*), okra (*Abelmoschus esculentus*), nag chapa (*Plumeria* sp.), rooster flower (*Celosia argentea*), yard long bean (*Vigna unguiculata*), garlic (*Allium sativum*), marigold (*Calendula arvensis*), lemon (*Citrus limon*), pumpkin (*Cucurbita moschata*), cucumber (*Cucumis sativus*), mustard (*Brassica rapa*), chili (*Capsicum annuum*), sweet potato (*Ipomoea batatas*), and tomato (*Solanum lycopersicum*) (Table S1). *Thrips palmi* was found in brinjal cucumber, pumpkin, marigold, bean, bitter gourd, ash gourd, ridge gourd, tomato, okra, and rose; *T. hawaiiensis* in bean, mustard, rose, marigold, and cotton; *T. parvispinus* in brinjal, chili, and marigold; *M. usitatus* in bean, brinjal, yard long bean, sponge gourd, bitter gourd, and rose; *T. florum* in bean, lemon, and rose; *F. intonsa* in rose, spider, brinjal, and pumpkin; *M. distalis* in mustard and rose; *Haplothrips* sp. in chili and cotton. *B. melanicornis* and *D. innoxius*, *M. abdominalis* and *A. chaetophora*, *T. tabaci*, *T. subnudula* and *S. dorsalis*, *Phlaeothripidae* sp., and *A. tumiceps* were found on sweet potatoes, marigold, garlic, chili, nag chapa, and roses, respectively. All species were polyphagous, and only one species, *Microcephalothrips abdominalis* is an important pollinator (Table 1).

### 3.2. Barcode Divergence of Thrips Species

In total, 207 mtCOI sequences representing 19 species were analyzed in the current study. The analysis revealed that the overall K2P/p-distance mean genetic distance of the dataset was 0.1719/0.1463. The intraspecific and interspecific distances (K2P/*p*-distance) showed substantial variation, ranging from 2.81/2.57%–47.51/35.18%, respectively (Table 2 and Table S2). Intraspecies distance could not be determined for four species (*T. subnudula*, *M. abdominalis*, *A. tumiceps*, and *B. melanicornis*) due to a single representative.

### 3.3. Genetic Diversity and Gene Flow Analysis

Variation in mtCOI gene structure was analyzed for 15 thrips species, excluding *Thrips subnudula*, *Microcephalothrips abdominalis*, *Astrothrips tumiceps*, and *Bathrips melanicornis* because only one sample was available. Genetic distances (Fst values) between all species ranged from 0 to 1.0 (Table 3). The number of segregation sites and haplotypes varied among different species, with *T. palmi* showing the highest diversity (14 haplotypes from 97 sequences). Other species exhibited fewer haplotypes, with some showing only one (Table 2). A population genetics study was conducted for this species. Tajima’s D statistical analysis revealed negative values in five species (*T. palmi*, *T. parvispinus*, *T. hawaiiensis*, *F. intonsa*, and *S. dorsalis*), and Fu’s Fs statistics for *T. palmi and F. intonsa* showed negative values with significant differences (Table 2). Negative Tajima’s D values, which represent an excess of low-frequency polymorphisms relative to expectation, are typically interpreted as demographic estimates of population growth or selection. Similarly, negative values of Fu’s Fs indicate an excess number of alleles.

**Figure 3:**
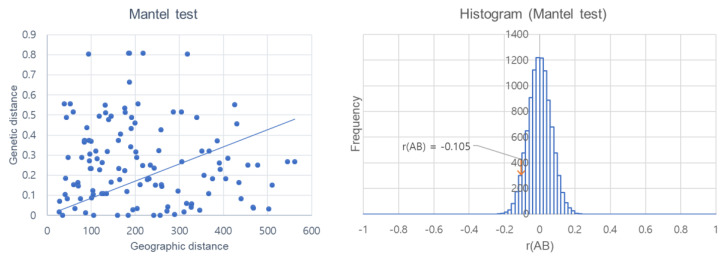
The relationship between the genetic distance (Fst) and geographic distance (km) of all samples of thrips species in Bangladesh. Each blue point represents population pairwise comparison Fst/(1-Fst) and histogram (Mantel test) shows the sampling distribution and orange arrow indicated the location of the observed correlation. The *p*-value was calculated using the r(AB) distribution estimated from 10,000 permutations.

**Figure 4:**
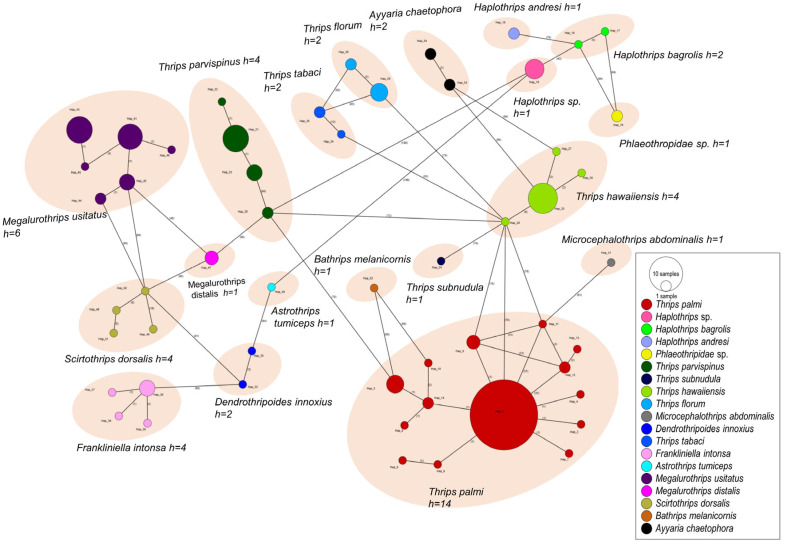
The evolutionary relationships of the identified haplotypes of thrips species, collected from 16 locations in Bangladesh, are depicted through minimum spanning networks. The areas of the circles are proportional to the frequency of each haplotype in the dataset. The color-coding represents the genetic groups of each haplotype.

The Mantel test revealed no correlation between genetic distance and geographic distance (r = −0.105, *p* = 0.101) (Figure 3). The minimum spanning network analysis was used to determine the evolutionary relationships among the haplotypes of each species. Haplotype networks derived from mtDNA sequences revealed a close relationship between all haplotypes (Figure 4). Nineteen thrips species were categorized into 54 haplotypes and were highly distant from each other by many mutational steps. The haplotype distribution reveals a clear graphical pattern encompassing 19 thrips species, each genetically unique from the others (Figure 4). Among them, *T. palmi* exhibits the highest diversity. Notably, Hap1 holds a central position in the network and is linked to 13 low-frequency singleton haplotypes. Hap 6, Hap 12, and Hap 14 of *T. palmi* represented two and three individual haplotypes, respectively. *Megalurothrips usitatus* and *T. parvispinus* have three dominantly represented haplotypes. *Thrips hawaiiensis*, *F. intonsa*, *S. dorsalis*, *T. tabaci*, *T. florum*, *A. chaetophora*, *D. innoxious*, and *H. bagrolis* have a single dominant haplotype with closely related 1–3 singletons. Other species showed a linear form of haplotypes (Figure 4). The minimum spanning network of haplotypes showed a similar pattern to the phylogenetic tree of the 19 thrips species. Hierarchical AMOVA indicated that most of the genetic variation (Fst = 97.7%) occurred within the population based on geographic distance (Table 4). Genetic variation among the groups was also high (68.31). The genetic variation was distributed as follows: 69.9% among groups, 0.85% among populations within groups, and 2.25% among populations.

## 4. Discussion

In this study, we examined the genetic diversity and performed DNA barcoding of thrips species in Bangladesh. This study provides the first DNA barcoding data to identify thrips species in Bangladesh. Additionally, this study contributed mtCOI sequences to the GenBank database and identified 19 species (Figure 2). We found that *T. palmi* was the dominant species, while *T. florum* was identified in only a few places. *T. palmi* and *T. hawaiiensis* distribution and population abundance were higher around the country, whereas only one or two individuals represented the other thrips species. For instance, *A. tumiceps* from Gazipur (Central), *A. chaetophora* from Rajshahi (West), *B. melanicornis*, *D. innoxious* and *T. subnudula* from Dinajpur (North), *H. bagrolis* from Rangpur (North), *T. tabaci* and *Haplothrips* sp. from Brahmanbaria (East), and *H. andresi*, *M. abdominalis* from Khulna (South) and *Phlaeothripidae* sp. from Khagrachori (Southeast). *T. Parvispinus*, *M. distalis*, *S. dorsalis*, and *F. intonsa* were found only in the northern regions of Bangladesh, where most of the thrips species were found (11 species). *M. usitatus* was abundant and widely distributed on the northern and southern sides. Most of the species included in this study are pests of various agricultural and horticultural crops.

An extensive survey of thrips species in Bangladesh showed that the pairwise mtCOI nucleotide sequence variation reached 35%, and 19 species were identified in samples collected from 16 different locations from 21 host plants during 2021–2023. Each species was genetically distinct. In our study, DNA sequencing of molecular studies has been conducted, and the results indicate clear genetic differences between the identified species (Figure 2 and Table 2). Species of thrips belonging to two suborders (Terebrantia and Tubulifera) and two families (Thripidae and Phlaeothripidae) were identified (Table 1). Most species were polyphagous, and only one was a pollinator [63]. Among the 19 species, *Dendrothripoides innoxius* was found in only one host plant from one location (Table 1). Similarly, Reyes [64] listed five species within the genus *Dendrothripoides*: one species native to South Africa, one to Thailand, two found in the Philippines, and one species commonly found on sweet potato plants worldwide. It is noteworthy that *T. palmi* was found to be more abundant than the other species. However, *F. occidentalis*, a more common vector species of TSWV, was not detected in this study. This species is absent in Bangladesh, even though it has invaded neighboring countries. In 2015, *F. occidentalis* was reported for the first time in India based on specimens collected from tomatoes [65]. Douglas [48] reported that *M. usitatus*, *M. distalis*, *T. palmi*, *T. hawaiiensis*, *F. intonsa*, Phlaeothripidae sp., and *Haplothrips* sp. were present only in bean host plants in Bangladesh. The BOLD database showed three species; *Azaleothrips* (GMBCA5994, GMBCM2508)*, Plicothrips apicalis* (GMBCD2954), and *Cephalothrips* (GMBCD2987) reported from southern Bangladesh that we did not find in our study. In this study, we found most thrips species on multiple hosts, including commercial and experimental fields in Bangladesh.

*T. palmi*, also known as the Melon thrips, is a common insect pest that affects vegetables and ornamentals [66]. There has been an increase in the prevalence of melon thrips in Southeast Asia, throughout most of the rest of Asia, Australia, North Africa, Central and South America, and the Caribbean [66,67]. The sequence divergence range of *T. palmi* was 0.2–6.8 (Table S3). The maximum K2P distance observed was 19.9% for *T. palmi*, 10.4% for *T. tabaci* [24]. Rebijith et al. [25] reported that 12.3% and 13.8% barcode divergence was found in *T. palmi and T. tabaci* from Indian populations. Furthermore, distance analysis revealed a maximum divergence of 13% in *T. palmi* and 12% in *T. tabaci* from Pakistan. Barcode gap analysis showed the maximum intraspecies distances (>2%) of *A. intermedius*, *H. reuteri*, *T. palmi* and *T. tabaci* [23]. A high maximum intraspecific distance indicates cryptic species within *T. palmi* populations [66]. Populations of *T. palmi* in various regions of Bangladesh exhibited 14 haplotypes. The genetic database of *T. palmi* has been expanded through numerous recent studies. A DNA polymorphism analysis of *T. palmi* populations worldwide uncovered 29 haplotypes. Phylogenetic analysis suggests that the *T. palmi* population can be categorized into three distinct lineages.

*T. parvispinus* (Karny) is a serious pest affecting several agricultural and horticultural crops in southeast Asia. In our study, *T. parvispinus* was found to be distributed on the northern side of Bangladesh in different host plants. Mound and Collins [68] reported that *T. parvispinus*, a member of the *T. orientalis* group, is present in Thailand and Australia. Our haplotype analysis showed that *T. parvispinus* has four haplotypes. In contrast, the Indian and Indonesian populations have two and three haplotypes, respectively [69]. In the case of *T. hawaiiensis*, it is widely distributed on various host plants in Bangladesh with four haplotypes. The thrips species *T. hawaiiensis* is a global pest of various plants, including the lookalike *T. florum*. Some of the damage attributed to *T. hawaiiensis* may actually be caused by *T. florum* [70]. The interspecific distance between *T. hawaiiensis* and *T. florum* was 15.0–15.5, and the genetic distance was 0.9847 in this study (Table 3).

Through the use of mtCOI molecular identification, we were able to identify two primary species of thrips in seven host plants: *M. usitatus* (14%) and *M. distalis* (1%). This forms the basis for future studies on the potential damage caused by these pests. The genetic distance between these two species is 0.9621 (Refer to Table 3). Thrips species that belong to the *Megalurothrips* genus are known to breed on Fabaceae plants. Both *M. usitatus* and *M. distalis* have a wide distribution across tropical Asia [71,72,73]. The distance analysis showed that the maximum divergence between these two species was <2% and six haplotypes clusters for *M. usitatus* and one haplotype for *M. distalis*.

The occurrence of other thrips species, *B. melanicornis, T. tabaci*, *A. chaetophora*, *T. subnudula*, *Haplothrips* sp., Phlaeothripidae sp., *S. dorsalis*, *F. intonsa*, and *A. tumiceps* are also concerning in Bangladesh. The barcode gap showed that the lowest intraspecies distance was found in *Haplothrips* sp., Phlaeothripidae sp., and *H. andresi* (Table 2). In this study, we identified *M. abdominalis* in marigolds. This species has been reported as a composite thrips and has been documented as an important pollinator of various asteracean plants [74,75]. However, damage to the petals and collars of *Asteraceae* is caused by heavy infestations of *M. abdominalis*. The pigmentation of the petals is lost, and seed development is hindered. They are known vectors of the tobacco streak virus in Parthenium weed (*Parthenium hysterophorus*) [76,77], blue mink (*Ageratum houstanianum*), and tobacco [78].

Kadirvel et al. [24] reported that *S. dorsalis* and *T. palmi* showed the highest intraspecific genetic variation, followed by *T. tabaci* and *F. occidentalis*. This suggests that the mtCOI gene could be a useful tool for categorizing different species and genera of thrips that coexist in specific crop environments. Our study found similar results, with the highest intraspecific genetic variation in *T. parvispinus* and *S. dorsalis*, followed by *T. tabaci* and *H. bagrolis*, using the mtCOI gene (Table 2). Species delimitation is often determined by the gap between the maximum intraspecific and minimum interspecific distances in various animal groups [79,80]. This method has been used to identify a complex group of snails using barcode gap analysis and several other species delimitation methods [81].

Our research revealed significant genetic differentiation among 19 thrips species in Bangladesh. However, the Mantel test results showed no significant correlation between genetic and geographic distances in thrips populations. Despite their weak flight capacity, thrips exhibit high gene flow between locations with long geographical distances, likely facilitated by human activity [82,83]. Li et al. [84] found that haplotype 2 was present in all populations of *T. tabaci*, suggesting that the dominance of haplotype 2 could be linked to the presence of insecticide-resistance genes. In our study, haplotype 1 was dominant in *T. plami* populations. Further research is needed to investigate the potential relationship between insecticide resistance and the population genetics of different species.

According to the present study, the presence of haplotypes referred to among the nineteen species showed a high degree of genetic variation. The COI barcoding-based identification gives huge advantages in that it is not constrained by developmental stages, nor the physical integrity of the samples collected. To better understand thrips population dynamics, we must understand the biology of thrips populations and determine their abundance and distribution over time, including the variation of economically important traits such as vector competency within and between populations.

## 5. Conclusions

Nineteen species were identified from the host plants sampled in this study. Notably, we identified ten thrips species that have not been previously reported in Bangladesh: *Thrips parvispinus*, *Bathrips melanicornis*, *Microcephalothrips abdominalis*, *Thrips florum*, *Ayyaria chaetophora*, *Thrips subnudula*, *Dendrothripoides innoxius*, *Astrothrips tumiceps, Scirtothrips dorsalis*, and *Thrips tabaci.* All species were polyphagous except *Microcephalothrips abdominalis*, an important pollinator of asteracean plants. We did not find *F. occidentalis* in our samples. This study contributes to a comprehensive understanding of genetic diversity and the distribution of thrips species in Bangladesh. DNA barcodes can be used to identify thrips species complexes and their genetic lineages. The consistent findings from various analyses, including DNA barcode, phylogenetic, and haplotype analyses, affirm the presence of 19 distinct thrips species within our dataset of 207 samples, providing robust support for the observed genetic differences reflecting genuine distinctions between species. In the future, the information generated here could be useful for monitoring changes in the diversity, abundance, and displacement of thrips populations. In addition, this is the first study about genetic diversity, DNA barcoding, and geographic distribution of different host plants in Bangladesh. Moreover, further research is necessary to fully comprehend the precise roles that these thrips populations play in the transmission of various orthotospoviruses and to develop effective management strategies.

## Figures and Tables

**Table 1:** Composition and status of all thrips species collected from 16 regions of Bangladesh on the different host plants during 2021–2023.

Family	Identified Species	Common Name	Status	Host Plants
Thripidae	*Thrips palmi* (47%)	Melon thrips	Polyphagous	Ash Gourd
				Bean
				Bitter gourd
				Brinjal
				Cucumber
				Marigold
				Okra
				Pumpkin
				Ridge gourd
				Rose
				Tomato
	*Thrips hawaiiensis* (9%)	The Hawaiian flower thrips	Highly polyphagous	Bean
				Cotton
				Marigold
				Mustard
				Rapeseed
	*Thrips parvispinus* (9%)	Taiwanese thrips	Polyphagous	Brinjal
				Chili
				Cucumber
				Marigold
	*Thrips florum* (3%)	Banana thrips	Highly polyphagous	Bean
				Cotton
				Lemon
				Rose
	*Thrips tabaci* (1%)	Onion thrips	Polyphagous	Garlic
	*Thrips subnudula* (one sample)	Flower thrips	Polyphagous	Chili
	*Ayyaria chaetophora* (2%)	-	Probably polyphagous	Marigold
	*Astrothrips tumiceps* (one sample)	-	Apparently polyphagous	Rose
	*Bathrips melanicornis* (one sample)	-	Polyphagous	Sweet potato
	*Dendrothripoides innoxius* (1%)	-	Monophagous	Sweet potato
	*Frankliniella intonsa* (3%)	Flower thrips	Polyphagous	Brinjal
				Pumpkin
				Rose
	*Megalurothrips usitatus* (14%)	Bean flower thrips	Oligophagous	Bean
				Bitter gourd
				Brinjal
				Rose
				Sponge gourd
				Yard long bean
	*Megalurothrips distalis* (1%)	Background bean thrips	-	Mustard
				Rose
	*Microcephalothrips abdominalis* (one sample)	Composite thrips	Important pollinator	Marigold
	*Scirtothrips dorsalis* (2%)	Chilli thrips	Highly polyphagous	Chili
Phlaeothripidae	*Haplothrips* sp. (3%)	-	Polyphagous	Chili
				Cotton
	*Haplothrips andresi* (1%)	-	Polyphagous	Rose
	*Haplothrips bagrolis* (1%)	-	Polyphagous	Rooster flower
	Phlaeothripidae (1%)	-	Polyphagous	Nag Chapa

**Table 2:** Genetic diversity and intraspecific distance of thrips species based on mtCOI gene.

Species	Number of Sequences	Intraspecific Distance		No. of Haplotype	Haplotype Diversity	Nucleotide Diversity	No. of Segregating Sites	Tajima D	Fu’s Fs Statistic
		*p*-Distance	K2P Distance						
*Thrips palmi*	97	0.0062	0.0065	14	0.38466	0.00579	43	−2.0927	−1.319
*Thrips parvispinus*	18	0.0257	0.0281	4	0.59477	0.02511	62	−1.1411	13.813
*Thrips hawaiiensis*	17	0.0024	0.0027	4	0.33088	0.00270	12	−2.2602	0.613
*Thrips florum*	7	0.0018	0.0022	2	0.47619	0.00182	2	0.0503	0.406
*Thrips tabaci*	3	0.0164	0.0169	2	0.66667	0.01657	13	0	4.053
*Frankliniella intonsa*	7	0.0019	0.0021	4	0.71429	0.00219	4	−1.4341	−1.217
*Megalurothrips usitatus*	29	0.0063	0.0068	6	0.73645	0.00677	9	1.7084	2.328
*Megalurothrips distalis*	3	0	0	1	0	0	0	0	0
*Scirtothrips dorsalis*	4	0.0247	0.0255	4	1.0000	0.02422	24	−0.4541	0.880
*Ayyaria chaetophora*	4	0.0022	0.0025	2	0.66667	0.00255	2	1.8930	1.530
*Haplothrips* sp.	6	0	0	1	0	0	0	0	0
*Haplothrips bagrolis*	2	0.0077	0.0078	2	1.00000	0.00956	5	0	1.609
*Haplothrips andresi*	2	0	0	1	0	0	0	0	0
Phlaeothripidae sp.	2	0	0	1	0	0	0	0	0
*Dendrothripoides* *innoxius*	2	0.0064	0.0065	2	1.00000	0.00574	3	0	1.386

**Table 3:** Pairwise genetic distance (Fst) among the thrips species from Bangladesh.

Species	*Thrips* *palmi*	*Thrips* *parvispinus*	*Thrips* *hawaiiensis*	*Thrips* *florum*	*Thrips* *tabaci*	*Frankliniella* *intonsa*	*Megalurothrips* *usitatus*	*Megalurothrips* *distalis*	*Scirtothrips* *dorsalis*	*Ayyaria* *chaetophora*	*Haplothrips* sp.	*Haplothrips* *bagrolis*	*Haplothrips* *andresi*	*Phlaeothripidae* sp.	*Dendrothripoides* *innoxius*
*Thrips palmi*	-														
*Thrips parvispinus*	0.9101	-													
*Thrips hawaiiensis*	0.9724	0.9048	-												
*Thrips florum*	0.9756	0.9196	0.9847	-											
*Thrips tabaci*	0.9331	0.8936	0.9413	0.9427	-										
*Frankliniella intonsa*	0.9800	0.9314	0.9869	0.9900	0.9503	-									
*Megalurothrips usitatus*	0.9645	0.9133	0.9715	0.9775	0.9398	0.9769	-								
*Megalurothrips distalis*	0.9847	0.9250	0.9922	0.9948	0.9583	0.9941	0.9621	-							
*Scirtothrips dorsalis*	0.9227	0.8739	0.9270	0.9351	0.8888	0.9334	0.9037	0.9230	-						
*Ayyaria chaetophora*	0.9803	0.9290	0.9859	0.9897	0.9534	0.9895	0.9758	0.9944	0.9386	-					
*Haplothrips* sp.	0.9900	0.9584	0.9956	0.9971	0.9732	0.9967	0.9894	1.0000	0.9612	0.9962	-				
*Haplothrips bagrolis*	0.9737	0.9441	0.9799	0.9818	0.9592	0.9822	0.9746	0.9841	0.9471	0.9818	0.9425	-			
*Haplothrips andresi*	0.9908	0.9595	0.9958	0.9971	0.9729	0.9967	0.9889	1.0000	0.9602	0.9964	1.0000	0.9645	-		
Phlaeothripidae sp.	0.9905	0.9592	0.9956	0.9972	0.9745	0.9966	0.9895	1.0000	0.9591	0.9962	1.0000	0.9740	1.0000	-	
*Dendrothripoides* *innoxius*	0.9671	0.9205	0.9769	0.9779	0.9406	0.9775	0.9672	0.9840	0.9070	0.9793	0.9907	0.9750	0.9909	0.9902	-

**Table 4:** Analysis of molecular variance (AMOVA) of thrips in Bangladesh at different hierarchical levels.

Source of Variation	df	Sum of Squares	Variance Components	Percentage of Variation	Fixation Indices (F-Statistics)
Among groups	18	10,885.224	68.317 Va	96.90	FCT = 0.969
Among populations within groups	31	142.789	0.602 Vb	0.85	FSC = 0.275
Within populations	161	255.075	1.584 Vc	2.25	FST = 0.977
Total	210	11,283.088	70.503	-	

## Data Availability

Datasets generated for this study can be obtained from the corresponding author upon request with proper justification.

## Supplementary Materials

The following supporting information can be downloaded at: https://www.mdpi.com/article/10.3390/insects15020107/s1, Figure S1. Pairwise nucleotide sequence identity color distance matrix for thrips species calculated by SDT v1.2.; Table S1. Information about the collection sites and host plants of thrips from Bangladesh.; Table S2. Inter-species divergence of thrips in Bangladesh, *p*-distance at lower left and Kimura 2 parameters at upper right, and intraspecific distances showed in the text.; Table S3. A pairwise comparison of COI sequences of thrips species from Bangladesh during 2021–2023.
