# Supplementary material for: Genetic Diversity and DNA Barcoding of Thrips in Bangladesh
Source: Insects. 2024 Feb 3;15(2):107. doi: 10.3390/insects15020107 (PMC10888972; doi:10.3390/insects15020107)
Supplement: Supplementary file 1 [file insects-15-00107-s001.zip › insects-2798455-supplementary.pdf]

Table S1. Information about the collection sites and host plants of thrips from Bangladesh

Sample no.	Collection date	Host Plant	Location	Name of species	Latitude and longitude	Accession number
Rang-Br-01	2021.11.17	*Solanum melongena*	Sankarpur, Badarganj, Rangpur	*Thrips palmi*	25° 40' 11.1288" N 89° 3' 19.764" E	OR481072
Rang-Br-09	2021.11.17	*Solanum melongena*	Palichora, Mithapukur, Rangpur	*Thrips palmi*	25° 44' 26.088" N 89° 15' 40.1004" E	OR481073
Dinaj-Br-11	2021.12.24	*Solanum melongena*	Pakerhut, Khansama, Dinajpur	*Thrips palmi*	25° 37' 18.8004" N 88° 38' 18.5604" E	OR481074
Nil-Be-15	2021.12.28	*Phaseolus vulgaris*	Kuthipara, Sadar, Nilphamari	*Thrips palmi*	25° 56' 35.232" N 89° 3' 48.744" E	OR481075
Lal-Br-18	2022.01.19	*Solanum melongena*	Kakina, Kaliganj, Lalmonirhut	*Thrips palmi*	23° 43' 15.996" N 88° 14' 24.864" E	OR481076
Panch-Be-26	2022.02.15	*Phaseolus vulgaris*	Daviganj, Panchagarh	*Thrips palmi*	26° 18' 47.7612" N 88° 31' 28.1964" E	OR481077
Panch-Pump-28	2022.02.15	*Cucurbita moschata*	Daviganj, Panchagarh	*Thrips palmi*	26° 18' 47.7612" N 88° 31' 28.1964" E	OR481078
Nil-Br-14	2021.12.28	*Solanum melongena*	Kuthipara, Sadar, Nilphamari	*Thrips palmi*	25° 56' 35.232" N 89° 3' 48.744" E	OR481079
Lal-Pump-20	2022.01.19	*Cucurbita moschata*	Biman Bandor, Sadar, Lalmonirhut	*Thrips palmi*	25° 44' 26.088" N 89° 15' 40.104" E	OR481080
Lal-Br-21	2022.01.19	*Solanum melongena*	Biman Bandor, Sadar, Lalmonirhut	*Thrips palmi*	25° 44' 26.088" N 89° 15' 40.104" E	OR481081
Kuri-Cucum-23	2022.01.19	*Cucumis sativus*	Singerdubri, Rajarhut, Kurigram	*Thrips palmi*	25° 44' 26.088" N 89° 15' 40.1004" E	OR481082
Kuri-Br-25	2022.01.19	*Solanum melongena*	Singerdubri, Rajarhut, Kurigram	*Thrips palmi*	25° 44' 26.088" N 89° 15' 40.1004" E	OR481083
Panch-Br-27	2022.02.15	*Solanum melongena*	Daviganj, Panchagarh	*Thrips palmi*	26° 18' 47.7612" N 88° 31' 28.1964" E	OR481084
Joy-Cucum-32	2022.01.27	*Cucumis sativus*	Panchbibi, Joypurhat	*Thrips palmi*	25° 12' 27.1332" N 89° 4' 25.7592" E	OR481085
Thakur-Pump-37	2022.04.06	*Cucurbita moschata*	Pirganj, Thakurgao	*Thrips palmi*	26° 1' 55.5636" N 88° 27' 38.5776" E	OR481086
Thakur-Ash-38	2022.04.06	*Benincasa hispida*	Pirganj, Thakurgao	*Thrips palmi*	26° 1' 55.5636" N 88° 27' 38.5776" E	OR481087
Bog-Ridge-44	2022.07.08	*Luffa acutangula*	Shantahar, Bogura	*Thrips palmi*	24° 48' 3.672" N	OR481088

					88° 59' 14.352" E	
Dinaj-Ash-46	2022.07.14	*Benincasa hispida*	Parbotipur, Dinajpur	*Thrips palmi*	25° 37' 18.804" N<br>88° 38' 18.564" E	OR481089
Bag-Br-Khul3	2022.02.05	*Solanum melongena*	Chitolmari, Bagerhat	*Thrips palmi*	22° 39' 32.616" N<br>89° 48' 25.236" E	OR481090
Bag-Mari-Khul4	2022.02.14	*Calendula arvensis*	Chitolmari, Bagerhat	*Thrips palmi*	22° 39' 32.616" N<br>89° 48' 25.236" E	OR481091
Gazi-Ro-G5	2022.12.22	*Rosa scinensis*	Gazipur	*Thrips palmi*	24° 5' 44.9448" N<br>90° 24' 45.0756" E	OR481092
Mymen-Br-M2	2022.08.07	*Solanum melongena*	Mymenshing	*Thrips palmi*	24° 44' 21.8292" N<br>90° 24' 28.7352" E	OR481093
Noa-Br-N2	2022.08.07	*Solanum melongena*	Noakhali	*Thrips palmi*	22° 48' 30.4956" N<br>91° 6' 59.0148" E	OR481094
Khag-Br-K1	2022.11.26	*Solanum melongena*	Khagrachori	*Thrips palmi*	23° 4' 19.8876" N<br>92° 0' 34.2324" E	OR481095
Rang-Br-B1	2023.01.01	*Solanum melongena*	Parbotipur Badarganj	*Thrips palmi*	25° 44' 26.088" N<br>89° 15' 40.104" E	OR481096
Khag-Ok-K7	2023.01.01	*Abelmoschus esculentus*	Khagrachori	*Thrips palmi*	23° 4' 19.8876" N<br>92° 0' 34.2324" E	OR481097
Brah-Br-1	2023.02.03	*Solanum melongena*	Brahmanbaria	*Thrips palmi*	23° 57' 50.9112" N<br>91° 7' 5.8836" E	OR481098
Rang-Mari-06	2021.11.17	*Calendula arvensis*	Soddopuscorni, Mithapukur, Rangpur	*Thrips palmi*	25° 44' 26.088" N<br>89° 15' 40.104" E	OR481099
Khag-T0-K4	2022.12.17	*Solanum lycopersicum*	Khagrachori	*Thrips palmi*	23° 4' 19.8876" N<br>92° 0' 34.2324" E	OR481100
Bog-Ash-42	2022.07.08	*Benincasa hispida*	Comarpur, Bogura	*Thrips palmi*	24° 48' 3.672" N<br>88° 59' 14.352" E	OR481101
Bog-Be-45	2022.07.08	*Phaseolus vulgaris*	Comarpur, Bogura	*Thrips palmi*	24° 48' 3.672" N<br>88° 59' 14.352" E	OR481102
Rang-Ridge-41	2022.04.06	*Luffa acutangula*	Kalamtol, Badarganj Rangpur	*Thrips palmi*	25° 44' 26.088" N<br>89° 15' 40.104" E	OR481103
Dinaj-Br-48	2022.07.14	*Solanum melongena*	Parbotipur dinajpur	*Thrips palmi*	25° 37' 18.804" N<br>88° 38' 18.564" E	OR481104
Dinaj-Pump-49	2022.07.14	*Cucurbita moschata*	Parbotipur dinajpur	*Thrips palmi*	25° 37' 18.804" N<br>88° 38' 18.564" E	OR481105
Rang-Bi-33	2022.03.04	*Momordica charantia*	Kalirhat, Badarganj Rangpur	*Thrips palmi*	25° 44' 26.088" N<br>89° 15' 40.104" E	OR481106

Khag-Mari-K6	2023.01.01	*Calendula arvensis*	Khagrachori	*Thrips palmi*	23° 4' 19.8876" N<br>92° 0' 34.2324" E	OR481107
Gazi-Ch-1	2022.12.22	*Capsicum annuum*	Gazipur	*Haplothrips sp.*	24° 5' 44.9448" N<br>90° 24' 45.0756" E	OR482070
Gazi-Co-1.3	2022.12.22	*Gossypium hirsutum*	Gazipur	*Haplothrips sp.*	24° 5' 44.9448" N<br>90° 24' 45.0756" E	OR482071
Brah-Ch-H1	2023.02.03	*Capsicum annuum*	Brahmanbaria	*Haplothrips sp*	23° 57' 50.9112" N<br>91° 7' 5.8836" E	OR482072
Rang-Ro-7.1	2021.11.17	*Celosia argentea*	Soddopuscorni, Mithapukur, Rangpur	*Haplothrips bagrolis*	25° 44' 26.088" N<br>89° 15' 40.104" E	OR482073
Rang-Ro-7.2	2021.11.17	*Celosia argentea*	Soddopuscorni, Mithapukur, Rangpur	*Haplothrips bagrolis*	25° 44' 26.088" N<br>89° 15' 40.104" E	OR482074
Bag-Ro-Khul9.1	2022.02.14	*Rosa scinensis*	Chitolmari, Bagerhat	*Haplothrips andresi*	22° 39' 32.616" N<br>89° 48' 25.236" E	OR482075
Bag-Ro-Khul9.2	2022.02.14	*Rosa scinensis*	Chitolmari, Bagerhat	*Haplothrips andresi*	22° 39' 32.616" N<br>89° 48' 25.236" E	OR482076
Khag-Nag-K2	2022.11.26	*Plumeria sp*	Khagrachori	*Phlaeothripidae sp*	23° 4' 19.8876" N<br>92° 0' 34.2324" E	OR482077
Khag-Nag-K2.3	2022.11.26	*Plumeria sp*	Khagrachori	*Phlaeothripidae sp*	23° 4' 19.8876" N<br>92° 0' 34.2324" E	OR482078
Khan-Mari-12	2021.12.24	*Calendula arvensis*	Pakerhut, Khansama, Dinajpur	*Thrips parvispinus*	25° 37' 18.8004" N<br>88° 38' 18.5604" E	OR482079
Khan-Mari-12.3	2021.12.24	*Calendula arvensis*	Pakerhut, Khansama, Dinajpur	*Thrips parvispinus*	25° 37' 18.8004" N<br>88° 38' 18.5604" E	OR482080
Joy-Cucum-32.1	2022.01.27	*Cucumis sativus*	Panchbibi, Joypurhat	*Thrips parvispinus*	25° 12' 27.1332" N<br>89° 4' 25.7592" E	OR482081
Lal-Ch-35	2022.04.28	*Capsicum annuum*	Naowdabash, Hatibandha, Lalmonirhat	*Thrips parvispinus*	25° 54' 58.9896" N<br>89° 26' 46.0356" E	OR482082
Rang-Br-1.4	2021.11.17	*Solanum melongena*	Sankarpur, Badarganj, Rangpur	*Thrips parvispinus*	25° 40' 11.1288" N<br>89° 3' 19.764" E	OR482083
Rang-Ch-3.4	2021.11.17	*Capsicum annuum*	Sankarpur, Badarganj, Rangpur	*Thrips parvispinus*	25° 40' 11.1288" N<br>89° 3' 19.764" E	OR482084
Joy-Ch-31	2022.01.27	*Capsicum annuum*	Panchbibi, Joypurhat	*Thrips parvispinus*	25° 12' 27.1332" N<br>89° 4' 25.7592" E	OR482085
Raj-Ch-R3.3	2022.07.23	*Capsicum annuum*	Rajshahi	*Thrips parvispinus*	24° 21' 48.9168" N<br>88° 37' 26.8968" E	OR482086

Rang-Br-C1	2023.01.01	*Solanum melongena*	Boirumpur, Badarganj	*Thrips parvispinus*	25° 44' 26.088" N<br>89° 15' 40.104" E	OR482087
Nil-Ch-34	2022.04.10	*Capsicum annuum*	Khuthipara sadar, Nilphamari	*Thrips parvispinus*	25° 56' 5.532" N<br>88° 51' 21.2544" E	OR482088
Dinaj-Ch-47	2022.07.14	*Capsicum annuum*	Parbotipur, Dinajpur	*Thrips subnudula*	25° 37' 18.804" N<br>88° 38' 18.564" E	OR482089
Rang-Be-2	2021.11.17	*Phaseolus vulgaris*	Sankarpur, Badarganj, Rangpur	*Thrips hawaiiensis*	25° 40' 11.1288" N<br>89° 3' 19.764" E	OR482090
Kuri-Mus-22	2022.01.19	*Brassica rapa*	Singerdubri, Rajarhut, Kurigram	*Thrips hawaiiensis*	25° 44' 26.088" N<br>89° 15' 40.1004" E	OR482091
Gazi-Co-T3	2022.06.12	*Gossypium hirsutum*	Gazipur	*Thrips hawaiiensis*	24° 5' 44.9448" N<br>90° 24' 45.0756" E	OR482092
Khag-Rap-K5	2023.01.01	*Brassica rapa*	Khagrachori	*Thrips hawaiiensis*	23° 4' 19.8876" N<br>92° 0' 34.2324" E	OR482093
Gazi-Co-1.4	2022.12.22	*Gossypium hirsutum*	Gazipur	*Thrips hawaiiensis*	24° 5' 44.9448" N<br>90° 24' 45.0756" E	OR482094
Gazi-Mari-F1	2022.12.22	*Calendula arvensis*	Gazipur	*Thrips hawaiiensis*	24° 5' 44.9448" N<br>90° 24' 45.0756" E	OR482095
Bag-Mari-Khul4	2022.02.14	*Calendula arvensis*	Chitolmari, Bagerhat	*Thrips hawaiiensis*	22° 39' 32.616" N<br>89° 48' 25.236" E	OR482096
Rang-Mari-6	2021.11.17	*Calendula arvensis*	Soddopuscorni, Mithapukur, Rangpur	*Thrips hawaiiensis*	25° 44' 26.088" N<br>89° 15' 40.104" E	OR482097
Lal-Be-19	2022.01.19	*Phaseolus vulgaris*	Kakina, Kaliganj, Lalmonirhut	*Thrips florum*	23° 43' 15.996" N<br>88° 14' 24.864" E	OR482098
Gazi-Co-T5	2022.12.22	*Gossypium hirsutum*	Gazipur	*Thrips florum*	24° 5' 44.9448" N<br>90° 24' 45.0756" E	OR482099
Rang-Ro-D1	2023.01.03	*Rosa scinensis*	Badarganj, Rangpur	*Thrips florum*	25° 44' 26.088" N<br>89° 15' 40.104" E	OR482100
Joy-Le-30	2022.01.27	*Citrus limon*	Panchbibi, Joypurhat	*Thrips florum*	25° 12' 27.1332" N<br>89° 4' 25.7592" E	OR482101
Bag-Mari-Khul4.3	2022.02.14	*Calendula arvensis*	Chitolmari, Bagerhat	*Microcephalothrips abdominalis*	22° 39' 32.616" N<br>89° 48' 25.236" E	OR482102
Dinaj-SP-A1.3	2022.12.04	*Ipomoea batatas*	Parbotipur Badarganj	*Dendrothripoides innoxius*	25° 44' 26.088" N<br>89° 15' 40.104" E	OR482103
Dinaj-SP-A1.4	2022.12.04	*Ipomoea batatas*	Parbotipur Badarganj	*Dendrothripoides innoxius*	25° 44' 26.088" N<br>89° 15' 40.104" E	OR482104
Brah-Ga-G1.2	2023.02.03	*Allium sativum*	Brahmanbaria	*Thrips tabaci*	23° 57' 50.9112" N	OR482105

					91° 7' 5.8836" E	
Brah-Ga-G1.3	2023.02.03	*Allium sativum*	Brahmanbaria	*Thrips tabaci*	23° 57' 50.9112" N<br>91° 7' 5.8836" E	OR482106
Brah-Ga-G1.4	2023.02.03	*Allium sativum*	Brahmanbaria	*Thrips tabaci*	23° 57' 50.9112" N<br>91° 7' 5.8836" E	OR482107
Rang-Ro-4.2	2021.11.17	*Rosa scinensis*	Soddopuscorni, Rangpur	*Frankliniella intonsa*	25° 44' 26.088" N<br>89° 15' 40.104" E	OR482108
Lal-Br-18.1	2022.01.19	*Solanum melongena*	Kakina, Kaliganj, Lalmonirhut	*Frankliniella intonsa*	23° 43' 15.996" N<br>88° 14' 24.864" E	OR482109
Lal-Pump-20.4	2022.01.19	*Cucurbita moschata*	Biman Bandor, Sadar, Lalmonirhut	*Frankliniella intonsa*	25° 44' 26.088" N<br>89° 15' 40.104" E	OR482110
Gazi-Ro-R1.3	2022.12.22	*Rosa scinensis*	Gazipur	*Astrothrips tumiceps*	24° 5' 44.9448" N<br>90° 24' 45.0756" E	OR482111
Brah-Be-1.2	2023.02.03	*Phaseolus vulgaris*	Brahmanbaria	*Megalurothrips usitaus*	23° 57' 50.9112" N<br>91° 7' 5.8836" E	OR482112
Rang-Bi-33.3	2022.03.04	*Momordica charantia*	Kalirhat, Badarganj Rangpur	*Megalurothrips uistatus*	25° 44' 26.088" N<br>89° 15' 40.104" E	OR482113
Nil-Yard-16	2021.12.28	Vigna unguiculata	Kuthipara, Sadar, Nilphamari	*Megalurothrips usitatus*	25° 56' 35.232" N<br>89° 3' 48.744" E	OR482114
Dinaj-Be-10	2021.12.24	*Phaseolus vulgaris*	Pakerhut, Khansama, Dinajpur	*Megalurothrips usitatus*	25° 37' 18.8004" N<br>88° 38' 18.5604" E	OR482115
Rang-Br-9	2021.11.17	*Solanum melongena*	Palichora, Mithapukur, Rangpur	*Megalurothrips usitatus*	25° 44' 26.088" N<br>89° 15' 40.1004" E	OR482116
Rang-Be-8	2021.11.17	*Phaseolus vulgaris*	Palichora, Mithapukur, Rangpur	*Megalurothrips usitatus*	25° 44' 26.088" N<br>89° 15' 40.1004" E	OR482117
Khag-Be-K3	2022.11.26	*Phaseolus vulgaris*	Khagrachori	*Megalurothrips usitatus*	23° 4' 19.8876" N<br>92° 0' 34.2324" E	OR482118
Rang-Be-8.1	2021.11.17	*Phaseolus vulgaris*	Palichora, Mithapukur, Rangpur	*Megalurothrips usitatus*	25° 44' 26.088" N<br>89° 15' 40.1004" E	OR482119
Rang-Ro-D1.1	2023.01.03	*Rosa scinensis*	Badarganj, Rangpur	*Megalurothrips usitatus*	25° 44' 26.088" N<br>89° 15' 40.104" E	OR482120
Nil-SG-17	2021.12.28	*Luffa cylindrica*	Kuthipara, Sadar, Nilphamari	*Megalurothrips usitatus*	25° 56' 35.232" N<br>89° 3' 48.744" E	OR482121
Lal-Be-19.1	2022.01.19	*Phaseolus vulgaris*	Kakina, Kaliganj, Lalmonirhut	*Megalurothrips usitatus*	23° 43' 15.996" N<br>88° 14' 24.864" E	OR482122
Nil-Be-15.3	2021.12.28	*Phaseolus vulgaris*	Kuthipara, Sadar, Nilphamari	*Megalurothrips usitatus*	25° 56' 35.232" N<br>89° 3' 48.744" E	OR482123

Rang-Be-2.4	2021.11.17	*Phaseolus vulgaris*	Sankarpur, Badarganj, Rangpur	*Megalurothrips usitatus*	25° 40' 11.1288" N 89° 3' 19.764" E	OR482124
Dinaj-Mus-13	2021.12.24	*Brassica rapa*	Pakerhut, Khansama, Dinajpur	*Megalurothrips distalis*	25° 37' 18.8004" N 88° 38' 18.5604" E	OR482125
Rang-Ro-D1.3	2023.01.03	*Rosa scinensis*	Badarganj, Rangpur	*Megalurothrips distalis*	25° 44' 26.088" N 89° 15' 40.104" E	OR482126
Dinaj-Ch-47.3	2022.07.14	*Capsicum annuum*	Parbotipur, Dinajpur	*Scirtothrips dorsalis*	25° 37' 18.804" N 88° 38' 18.564" E	OR482127
Dinaj-Ch-47.4	2022.07.14	*Capsicum annuum*	Parbotipur, Dinajpur	*Scirtothrips dorsalis*	25° 37' 18.804" N 88° 38' 18.564" E	OR482128
Joy- Ch-31.3	2022.01.27	*Capsicum annuum*	Panchbibi, Joypurhat	*Scirtothrips dorsalis*	25° 12' 27.1332" N 89° 4' 25.7592" E	OR482129
Dinaj-SP-A1.1	2022.12.04	*Ipomoea batatas*	Parbotipur Badarganj	*Bathrips melanicornis*	25° 44' 26.088" N 89° 15' 40.104" E	OR482130
Raj-Mari-R2.1	2022.07.23	*Calendula arvensis*	Rajshahi	*Ayyaria chaetophora*	24° 21' 48.9168" N 88° 37' 26.8968" E	OR482131
Raj-Ch-R2.3	2022.07.23	*Calendula arvensis*	Rajshahi	*Ayyaria chaetophora*	24° 21' 48.9168" N 88° 37' 26.8968" E	OR482132
Raj-Ch-R3.1	2022.07.23	*Calendula arvensis*	Rajshahi	*Ayyaria chaetophora*	24° 21' 48.9168" N 88° 37' 26.8968" E	OR482133

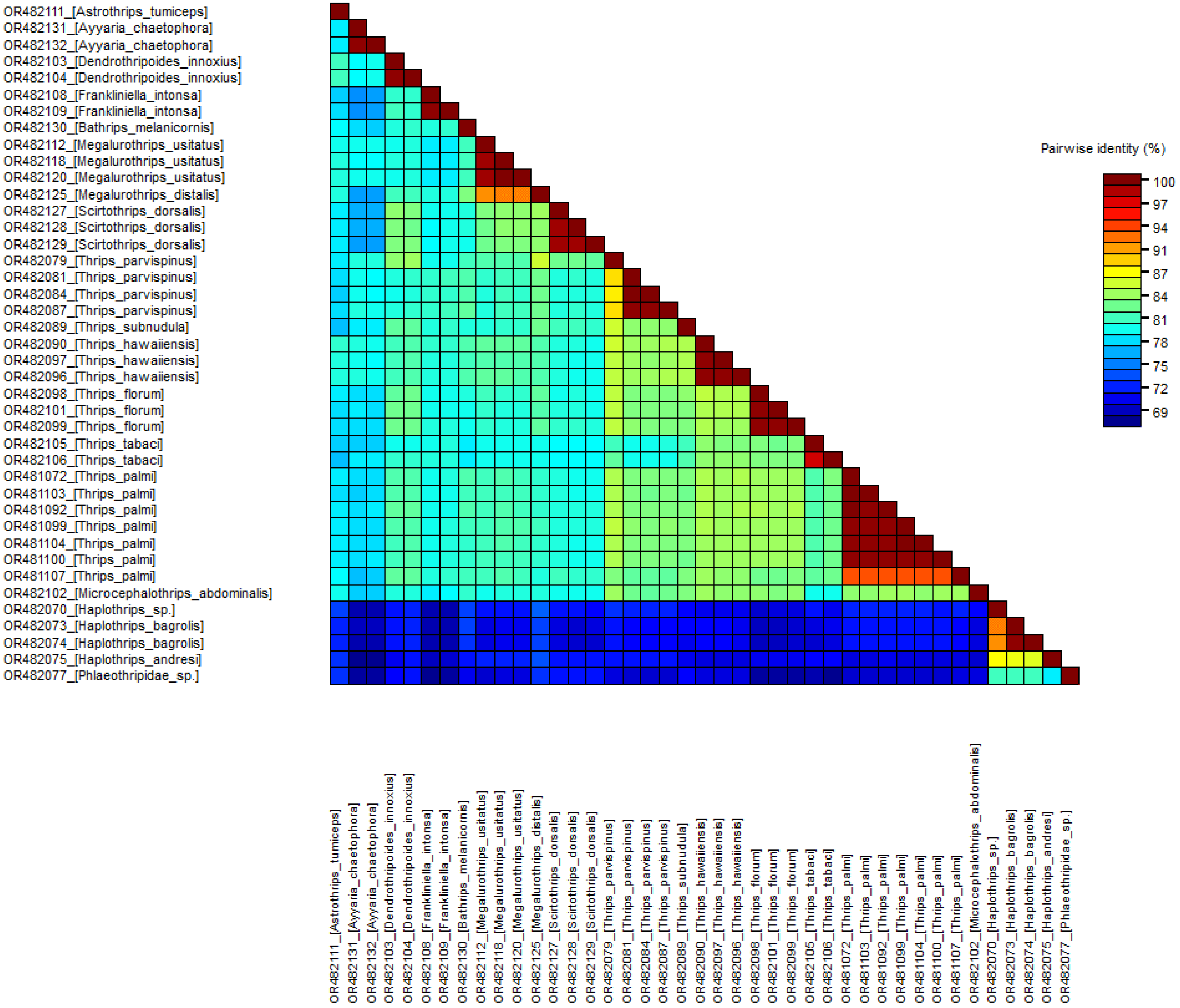

Figure S1. Pairwise nucleotide sequences identity color distance matrix for thrips species calculated by SDT v1.2.

Table S2. Inter-species divergence of thrips in Bangladesh, p-distance at lower left and Kimura 2 parameters at upper right and intraspecific distances showed in the text

Species	*Thrips palmi*	*Thrips parvispinus*	*Thrips hawaiiensis*	*Thrips florum*	*Thrips tabaci*	*Thrips subnudula*	*Frankliniella intonsa*	*Megalurothrips usitatus*	*Megalurothrips distalis*	*Scirtothrips dorsalis*	*Ayyaria chaetophora*	*Haplothrips* sp.	*Haplothrips bagrolis*	*Haplothrips andresi*	*Phlaeothripidae* sp.	*Dendrothripoides innoxius*	*Microcephalothrips abdominalis*	*Astrothrips tumiceps*	*Bathrips melanicornis*
Thrips palmi	-	0.1958	0.1726	0.1754	0.1902	0.1868	0.2328	0.2016	0.2184	0.2247	0.2510	0.3651	0.3689	0.4070	0.3895	0.1996	0.1811	0.2364	0.1984
Thrips parvispinus	0.1718	-	0.1634	0.1904	0.2286	0.1814	0.2334	0.2113	0.1897	0.2269	0.2265	0.3859	0.4008	0.3995	0.3955	0.2251	0.1915	0.2399	0.2005
Thrips hawaiiensis	0.1539	0.1461	-	0.1655	0.1857	0.1665	0.2149	0.1875	0.1958	0.2119	0.2140	0.3931	0.3902	0.4127	0.3969	0.2096	0.1791	0.2213	0.1962
Thrips florum	0.1557	0.1674	0.1480	-	0.1820	0.1935	0.2338	0.2205	0.2006	0.2337	0.2507	0.4030	0.4045	0.3996	0.4353	0.1937	0.1842	0.2366	0.2365
Thrips tabaci	0.1672	0.1959	0.1640	0.1606	-	0.2121	0.2190	0.2244	0.2315	0.2111	0.2405	0.3987	0.4176	0.3932	0.4263	0.2164	0.2361	0.2550	0.2399
Thrips subnudula	0.1646	0.1605	0.1489	0.1702	0.1836	-	0.2123	0.2204	0.1958	0.2166	0.2322	0.3963	0.3915	0.4094	0.3739	0.2044	0.1809	0.2576	0.2265
Frankliniella intonsa	0.1992	0.1989	0.1865	0.1994	0.1888	0.1844	-	0.2241	0.2143	0.2314	0.2701	0.4310	0.4339	0.4347	0.4177	0.2012	0.2392	0.2429	0.2200
Megalurothrips usitatus	0.1767	0.1839	0.1659	0.1909	0.1939	0.1903	0.1936	-	0.0952	0.1812	0.2229	0.4159	0.4204	0.3923	0.4223	0.2202	0.2349	0.2419	0.2000
Megalurothrips distalis	0.1895	0.1673	0.1723	0.1759	0.1989	0.1721	0.1860	0.0893	-	0.1765	0.2690	0.3651	0.3837	0.3682	0.3713	0.2042	0.2239	0.2363	0.1986
Scirtothrips dorsalis	0.1940	0.1956	0.1844	0.2007	0.1834	0.1879	0.1982	0.1610	0.1573	-	0.2579	0.4029	0.4161	0.3899	0.3771	0.1814	0.2254	0.2402	0.2253
Ayyaria chaetophora	0.2120	0.1947	0.1859	0.2128	0.2052	0.1989	0.2262	0.1926	0.2256	0.2180	-	0.4380	0.4387	**0.4751**	0.4365	0.2332	0.2481	0.2289	0.2327
Haplothrips sp.	0.2891	0.3016	0.3059	0.3117	0.3091	0.3078	0.3278	0.3190	0.2887	0.3117	0.3308	-	0.0887	0.1498	0.2161	0.3980	0.4064	0.3587	0.3866
Haplothrips bagrolis	0.2913	0.3103	0.3042	0.3125	0.3199	0.3050	0.3294	0.3218	0.3002	0.3193	0.3317	0.0832	-	0.1486	0.2113	0.3931	0.4229	0.3710	0.3931
Haplothrips andresi	0.3142	0.3096	0.3174	0.3098	0.3059	0.3155	0.3297	0.3051	0.2906	0.3040	**0.3518**	0.1358	0.1348	-	0.2579	0.4114	0.4195	0.3905	0.4061
Phlaeothripidae sp.	0.3038	0.3073	0.3082	0.3302	0.3250	0.2945	0.3199	0.3225	0.2925	0.2964	0.3308	0.1874	0.1836	0.2180	-	0.3708	0.4127	0.3771	0.4228
Dendrothripoides innoxius	0.1751	0.1940	0.1827	0.1706	0.1877	0.1788	0.1759	0.1907	0.1788	0.1611	0.1998	0.3088	0.3059	0.3164	0.2925	-	0.2227	0.2067	0.2224
Microcephalothrips abdominalis	0.1603	0.1684	0.1591	0.1625	0.2008	0.1606	0.2032	0.2016	0.1931	0.1946	0.2103	0.3136	0.3231	0.3212	0.3174	0.1922	-	0.2445	0.2073
Astrothrips tumiceps	0.2027	0.2051	0.1915	0.2027	0.2161	0.2180	0.2073	0.2068	0.2027	0.2055	0.1969	0.2849	0.2925	0.3040	0.2964	0.1807	0.2084	-	0.2393
Bathrips melanicornis	0.1740	0.1752	0.1723	0.2019	0.2046	0.1950	0.1898	0.1752	0.1740	0.1941	0.1989	0.3021	0.3059	0.3136	0.3231	0.1922	0.1797	0.2046	-

Table S3. A pairwise comparison of COI sequences of thrips species from Bangladesh during 2021-2023

	OR481072_Thrips_palmi	OR481092_Thrips_palmi	OR481099_Thrips_palmi	OR481100_Thrips_palmi	OR481103_Thrips_palmi	OR481104_Thrips_palmi	OR481107_Thrips_palmi	OR482070_Haplothrips_sp.	OR482073_Haplothrips_bagrolis	OR482074_Haplothrips_bagrolis	OR482075_Haplothrips_andresi	OR482077_Phlaeothripidae_sp.	OR482079_Thrips_parvispinus	OR482081_Thrips_parvispinus	OR482084_Thrips_parvispinus	OR482087_Thrips_parvispinus	OR482089_Thrips_subnudula	OR482090_Thrips_hawaiiensis	OR482096_Thrips_hawaiiensis	OR482097_Thrips_hawaiiensis	OR482098_Thrips_florum	OR482099_Thrips_florum	OR482101_Thrips_florum	OR482102_Microcephalothrips_abdominalis	OR482103_Dendrothripoides_innoxius	OR482104_Dendrothripoides_innoxius	OR482105_Thrips_tabaci	OR482106_Thrips_tabaci	OR482108_Frankliniella_intonsa	OR482109_Frankliniella_intonsa	OR482111_Astrothrips_tumiceps	OR482112_Megalurothrips_usitatus	OR482118_Megalurothrips_usitatus	OR482120_Megalurothrips_usitatus	OR482125_Megalurothrips_distalis	OR482127_Scirtothrips_dorsalis	OR482128_Scirtothrips_dorsalis	OR482129_Scirtothrips_dorsalis	OR482130_Bathrips_melanicornis	OR482131_Ayyaria_chaetophora	OR482132_Ayyaria
OR481072_Thrips_palmi																																									
OR481092_Thrips_palmi	0.002																																								
OR481099_Thrips_palmi	0.004	0.005																																							
OR481100_Thrips_palmi	0.005	0.007	0.005																																						
OR481103_Thrips_palmi	0.002	0.004	0.005	0.007																																					
OR481104_Thrips_palmi	0.005	0.007	0.002	0.007	0.007																																				
OR481107_Thrips_palmi	0.063	0.063	0.065	0.068	0.065	0.067																																			
OR482070_Haplothrips_sp.	0.301	0.303	0.301	0.305	0.303	0.301	0.308																																		
OR482073_Haplothrips_bagrolis	0.303	0.305	0.303	0.303	0.305	0.303	0.314	0.079																																	
OR482074_Haplothrips_bagrolis	0.303	0.305	0.303	0.303	0.305	0.303	0.314	0.081	0.009																																
OR482075_Haplothrips_andresi	0.328	0.326	0.328	0.332	0.330	0.328	0.344	0.132	0.132	0.133																															
OR482077_Phlaeothripidae_sp.	0.324	0.323	0.321	0.324	0.326	0.321	0.328	0.187	0.182	0.182	0.214																														
OR482079_Thrips_parvispinus	0.153	0.151	0.150	0.153	0.155	0.151	0.168	0.294	0.306	0.306	0.308	0.317																													
OR482081_Thrips_parvispinus	0.169	0.168	0.166	0.169	0.171	0.168	0.184	0.308	0.315	0.315	0.319	0.321	0.119																												
OR482084_Thrips_parvispinus	0.171	0.169	0.168	0.171	0.173	0.169	0.186	0.310	0.317	0.317	0.321	0.323	0.121	0.002																											
OR482087_Thrips_parvispinus	0.168	0.166	0.164	0.168	0.169	0.166	0.182	0.306	0.314	0.314	0.317	0.319	0.117	0.002	0.004																										
OR482089_Thrips_subnudula	0.162	0.160	0.160	0.164	0.164	0.162	0.168	0.319	0.312	0.314	0.326	0.310	0.144	0.162	0.164	0.160																									
OR482090_Thrips_hawaiiensis	0.157	0.157	0.159	0.162	0.159	0.160	0.166	0.317	0.312	0.312	0.326	0.315	0.139	0.155	0.157	0.153	0.151																								
OR482096_Thrips_hawaiiensis	0.160	0.160	0.162	0.166	0.162	0.164	0.169	0.319	0.314	0.314	0.328	0.317	0.142	0.155	0.157	0.153	0.151	0.004																							
OR482097_Thrips_hawaiiensis	0.160	0.160	0.162	0.166	0.162	0.164	0.169	0.315	0.310	0.310	0.324	0.319	0.142	0.159	0.160	0.157	0.151	0.004	0.007																						
OR482098_Thrips_florum	0.151	0.150	0.151	0.153	0.153	0.153	0.171	0.324	0.323	0.323	0.323	0.341	0.144	0.168	0.169	0.169	0.168	0.150	0.153	0.153																					
OR482099_Thrips_florum	0.151	0.150	0.151	0.153	0.153	0.153	0.171	0.324	0.319	0.319	0.323	0.339	0.142	0.171	0.173	0.173	0.168	0.151	0.155	0.155	0.004																				
OR482101_Thrips_florum	0.153	0.151	0.153	0.155	0.155	0.155	0.173	0.323	0.321	0.321	0.321	0.339	0.144	0.169	0.171	0.171	0.169	0.150	0.153	0.153	0.002	0.005																			
OR482102_Microcephalothrips_abdominalis	0.159	0.157	0.157	0.155	0.160	0.159	0.155	0.326	0.332	0.332	0.332	0.326	0.155	0.169	0.171	0.168	0.162	0.155	0.159	0.159	0.162	0.162	0.164																		
OR482103_Dendrothripoides_innoxius	0.180	0.178	0.182	0.186	0.182	0.184	0.177	0.308	0.303	0.305	0.319	0.303	0.160	0.200	0.202	0.198	0.175	0.182	0.186	0.182	0.173	0.171	0.173	0.195																	
OR482104_Dendrothripoides_innoxius	0.178	0.177	0.180	0.184	0.180	0.182	0.182	0.310	0.301	0.303	0.321	0.299	0.159	0.198	0.200	0.196	0.180	0.180	0.184	0.180	0.175	0.173	0.175	0.200	0.005																
OR482105_Thrips_tabaci	0.171	0.173	0.173	0.175	0.173	0.173	0.169	0.314	0.326	0.330	0.314	0.335	0.184	0.207	0.209	0.205	0.191	0.162	0.166	0.166	0.169	0.169	0.169	0.202	0.193	0.195															
OR482106_Thrips_tabaci	0.169	0.171	0.171	0.173	0.171	0.171	0.169	0.321	0.324	0.328	0.314	0.337	0.177	0.204	0.205	0.202	0.184	0.162	0.166	0.166	0.164	0.164	0.164	0.196	0.187	0.189	0.025														
OR482108_Frankliniella_intonsa	0.205	0.207	0.204	0.205	0.207	0.205	0.205	0.341	0.337	0.337	0.342	0.335	0.202	0.204	0.205	0.202	0.191	0.193	0.196	0.196	0.205	0.205	0.207	0.207	0.182	0.186	0.198	0.198													
OR482109_Frankliniella_intonsa	0.204	0.205	0.202	0.204	0.205	0.204	0.202	0.337	0.335	0.335	0.339	0.333	0.198	0.200	0.202	0.198	0.187	0.191	0.195	0.195	0.202	0.202	0.204	0.204	0.178	0.184	0.195	0.195	0.004												
OR482111_Astrothrips_tumiceps	0.204	0.204	0.200	0.204	0.205	0.200	0.204	0.290	0.294	0.294	0.306	0.301	0.198	0.204	0.205	0.202	0.220	0.186	0.189	0.189	0.205	0.205	0.207	0.207	0.191	0.189	0.209	0.216	0.207	0.204											
OR482112_Megalurothrips_usitatus	0.177	0.177	0.175	0.178	0.178	0.175	0.184	0.319	0.323	0.323	0.308	0.335	0.173	0.187	0.189	0.189	0.193	0.173	0.173	0.177	0.189	0.189	0.191	0.207	0.193	0.195	0.195	0.196	0.200	0.196	0.211										
OR482118_Megalurothrips_usitatus	0.173	0.173	0.171	0.175	0.175	0.171	0.180	0.317	0.319	0.319	0.310	0.330	0.171	0.180	0.182	0.178	0.187	0.171	0.171	0.175	0.195	0.195	0.196	0.198	0.189	0.191	0.195	0.196	0.200	0.196	0.205	0.013									
OR482120_Megalurothrips_usitatus	0.175	0.175	0.173	0.177	0.177	0.173	0.182	0.319	0.321	0.321	0.310	0.332	0.173	0.182	0.184	0.180	0.189	0.173	0.173	0.177	0.195	0.195	0.196	0.200	0.191	0.193	0.195	0.196	0.200	0.196	0.205	0.011	0.002								
OR482125_Megalurothrips_distalis	0.187	0.187	0.184	0.187	0.189	0.184	0.195	0.285	0.292	0.292	0.292	0.297	0.132	0.169	0.171	0.168	0.169	0.175	0.177	0.178	0.175	0.175	0.177	0.195	0.178	0.180	0.200	0.198	0.187	0.184	0.198	0.092	0.086	0.088							
OR482127_Scirtothrips_dorsalis	0.193	0.191	0.191	0.193	0.195	0.193	0.187	0.310	0.312	0.315	0.308	0.303	0.168	0.200	0.202	0.198	0.187	0.189	0.189	0.193	0.202	0.200	0.204	0.200	0.160	0.162	0.193	0.196	0.198	0.195	0.209	0.157	0.151	0.153	0.153						
OR482128_Scirtothrips_dorsalis	0.198	0.196	0.196	0.198	0.200	0.198	0.195	0.315	0.319	0.323	0.314	0.306	0.169	0.198	0.200	0.196	0.187	0.195	0.195	0.198	0.202	0.200	0.204	0.200	0.166	0.168	0.195	0.198	0.205	0.202	0.216	0.162	0.153	0.155	0.157	0.011					
OR482129_Scirtothrips_dorsalis	0.198	0.196	0.196	0.198	0.200	0.198	0.193	0.315	0.317	0.321	0.312	0.306	0.175	0.202	0.204	0.200	0.189	0.196	0.196	0.200	0.205	0.204	0.207	0.200	0.164	0.166	0.195	0.198	0.204	0.200	0.214	0.164	0.155	0.157	0.160	0.009	0.009				
OR482130_Bathrips_melanicornis	0.184	0.182	0.180	0.180	0.186	0.180	0.184	0.308	0.310	0.314	0.321	0.335	0.177	0.175	0.177	0.173	0.196	0.175	0.175	0.178	0.202	0.205	0.202	0.193	0.196	0.198	0.207	0.205	0.191	0.187	0.209	0.178	0.178	0.177	0.175	0.187	0.191	0.195			
OR482131_Ayyaria_chaetophora	0.209	0.207	0.207	0.207	0.211	0.207	0.214	0.326	0.326	0.328	0.350	0.335	0.189	0.191	0.193	0.189	0.198	0.187	0.187	0.191	0.211	0.214	0.209	0.216	0.205	0.200	0.205	0.204	0.232	0.231	0.204	0.187	0.191	0.189	0.222	0.218	0.223	0.225	0.204		
OR482132_Ayyaria_chaetophora	0.205	0.204	0.204	0.204	0.207	0.204	0.211	0.326	0.326	0.328	0.350	0.335	0.189	0.195	0.196	0.193	0.198	0.191	0.191	0.195	0.214	0.214	0.213	0.213	0.202	0.196	0.205	0.204	0.229	0.227	0.204	0.187	0.191	0.189	0.222	0.218	0.223	0.225	0.207	0.004	
